# Postpartum Maternal Morbidity Requiring Hospital Admission in A Teaching Hospital: A Descriptive Cross-sectional Study

**DOI:** 10.31729/jnma.5125

**Published:** 2020-09-30

**Authors:** Pravin Shrestha, Vibha Mahato, Smita Shrestha Karmacharya

**Affiliations:** 1 Department of Obstetrics and Gynecology, Manipal College of Medical Sciences, Pokhara, Nepal; 2 Nobel College, Sinamangal, Kathmandu, Nepal

**Keywords:** *hospital admission*, *maternal morbidity*, *postpartum*

## Abstract

**Introduction:**

Major concern shifts from mother to newborn in postnatal period. Postpartum complications contribute to a lot of maternal morbidity and mortality. This study aims to determine the prevalence of morbidities in women following delivery at Manipal Teaching Hospital so as to identify and improve maternal quality care.

**Methods:**

This is a descriptive cross-sectional study conducted at department of Obstetrics and Gynaecology, Manipal Teaching Hospital from September 2018 to March 2020 after ethical approval from the institutional review committee with reference number 1296. All the women presenting to the department during the study period were included in the study. Women who were admitted to accompany and nurse their babies for neonatal problems were excluded. Point estimate at 95% Confidence Interval was calculated along with frequency and proportion for binary data. Data were entered in Excel and analysed in SPSS.

**Results:**

Among 3510 cases, 104 women were admitted with various postpartum morbidities. The prevalenceof postpartum morbidity was found to be 104 (2.96%) at 95% Confidence Interval (2.67-3.25). Puerperal sepsis was diagnosed in 23 (22.11%), preeclampsia in 20(19.23%) eclampsia in 14 (13.46%) and haemorrhage in 14 (13.46%) respectively. Majority of patients, 83.65% belonged to age group of 20-34 years. Nine patients (8.65%) were teenage mothers.

**Conclusions:**

Puerperal sepsis, preeclampsia, eclampsia and haemorrhage were the major postpartum complications requiring admissions in hospital.

## INTRODUCTION

Postnatal care is an important part of maternal care as life-threatening complications can occur in the postpartum period. Reducing maternal mortality is a priority agenda of the national and international community as evidenced by the great interest in the Millennium Development Goal (MDG).^1^ In both developing and developed countries >60% of maternal deaths occurred in the postpartum period.^2^

According to Nepal demographic health survey, only about 45% of Nepali women receive postnatal care in the first seven days after delivery.^3^ The incidence of maternal readmission for postpartum morbidity was 1.25%, infectious morbidity being the commonest one.^4^

Maternal morbidity is extensive and under-recognised after delivery. How often these complications occur and when women seek health care is not very well documented. In present study an attempt has been made to determine the prevalence of morbidities in postpartum period so as to identify points of intervention for quality improvement in maternal care.

## METHODS

This is a descriptive cross-sectional study conducted in the department of Obstetrics and Gynaecology Manipal Teaching Hospital, Pokhara, Nepal from September 2018 to March 2020. The study was conducted after ethical approval from the institutional review committee with reference number 1296 and consent from patients.

The WHO definition of the postpartum period as beginning one hour after the delivery of placenta continuing till six weeks after the birth of the baby,^5^ was used as the time period for inclusion criteria in this study. All women (3510) presenting to the department of Obstetrics and Gynaecology during the study period were included in the study (census sampling). All patients, either delivered in the study hospital or delivered elsewhere admitted with postpartum morbidity were included. Patients who were admitted just to accompany and nurse their babies for neonatal problems were excluded. The study mainly focussed on types of morbidities, postpartum day of admission and duration of hospital stay.

The data collected were recorded in a predesigned proforma and entered in excel sheet. SPSS (Statistical package for social science 16) was used for calculation and tabulation of data. The final results were discussed and conclusion was derived.

## RESULTS

A total of 104 women presented with various postpartum morbiditiesduring the study period. Total obstetric admissions were 3510 during the study period. The prevalence of postpartum admission was 2.96% at 95% Confidence Interval (2.67-3.25). The age ranges from 16 to 40 years with a mean of 26.31(SD 5.3). Majority of patients, 83.65% belonged to age group of 20-34 years. Nine patients (8.65%) were teenage mothers. Fifty seven (54.80%) patients admitted with postpartum morbidity had caesarean delivery as shown in (Table 1).

**Table 1:** Demographic characteristics and health variables influencing postpartum morbidity (n = 104).

Variable	n %
Age of the mother (years)	
<20	9 (8.65)
20-34	87 (83.65)
>34	8 (7.69)
Period of gestation	
<37 weeks	10 (9.61)
≥37 weeks	94 (90.38)
Parity	
Primipara	61 (58.6)
Multipara	43 (41.3)
Mode of delivery	
Vaginal	47 (45.1)
Caesarean section	57 (54.8)

Twelve women (11.53%) were admitted within two days of delivery, 49 (47.11%) in 2-7 days and 43 (41.34%) after 7 days for various postpartum morbidity. The duration of hospital stay in this study was less than 7 days in 87 (83.65%) patients and more than 7days for 17 (16.34%) patients. The average duration of hospital stay was 5.98 days (range 2-18).

Puerperal sepsis was diagnosed in 23 (22.11%) patients, preeclampsia in 20 (19.23%) patients and eclampsia in 14 (13.46%) patients. Six among them had delivered in the study hospital and eight patients were referred. Fourteen (13.46%) patients presented with postpartum haemorrhage. Out of total 14 patients, 6 patients had surgical evacuation, one patient underwent hysterectomy for placenta accreta and others were managed conservatively. In this study 8 patients (7.69%) had urinary tract infection, of them 3 presented with retention of urine.

**Figure 1:**
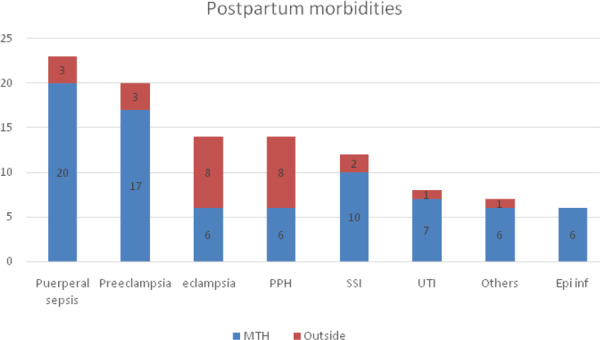
Distribution of postpartum morbidities

Seven (6.73%) patients presented with other complications which included postpartum cardiomyopathy in 2 patients (1.92%), paroxysomal supraventricular tachycardia (PSVT) in 1 (0.96%) patient (who were managed in ICU with cardiology team), organic psychosis in 1 (0.96%) patient, rectus sheath hematoma in 1 (0.96%) patient (who underwent exploration) and 2 (1.92%) patients with abdominal distension who were managed conservatively. Distribution of postpartum morbidity booked in study hospital and referred from other places has been shown (Figure 1).

Total obstetric admissions were 3510 during the study period of which 2988 (85.13%) patients delivered at the study hospital. The prevalence of postpartum morbidity was 104 (2.96%) among which 78 (75%) patients had their delivery in the study hospital. Of these 78 patients, 27 (34.62%) patients had vaginal delivery and 51 (65.39%) had caesarean section.

## DISCUSSION

The period after delivery represents a source of significant morbidity and mortality for women and their babies. In this study incidence of postpartum morbidity was 2.96 % which was similar to the study done by Raut B, et al. 1.94% and Malla R, et al. 1.58%.^4,7^ The mean age in our study was 26.31 years which was similar to the study done by Malla R, et al. with mean age of 26.7 years.^7^

Among patients admitted with postpartum morbidities the duration of hospital stay was 2-7 days for 83.65% and >7days for 16.34%. The average duration of hospital stay was 5.98 days (range 2-18) which was similar to the study done by Raut B^6^ where 73.8% were admitted for 3-7 days and average duration of hospital stay was 4.53 days.

In this study puerperal sepsis was diagnosed in 22.11%. Pueperal sepsis was the most common complication in the study done by Vallely L, et al. in Lusaka, Zambia 34.8%.^8^ Hypertension and eclampsia accounts for 12% of maternal mortality.^9^ In our study, 19.23% women were admitted for preeclampsia and 13.46% with eclampsia. Women admitted with hypertensive disorder was higher in our study as compared to study done by Raut B 8.8%.^4^ The higher incidence of hypertensive disorder in our study could be due to the inclusion of patients who were referred from other centres as well. A study done by Rana S, et al. showed approximately 15% of postpartum eclampsia.^10^ Ensuring a proper antenatal and postnatal care is important in seeking timely care which can reduce maternal mortality.

About 13.46% patients presented with postpartum haemorrhage in present study. Out of these 14 patients, 6 patients had surgical evacuation, 1 patient underwent hysterectomy for placenta accreta and others were managed conservatively. 28.57% required blood transfusion. Postpartum haemorrhage was found in 22.8% of women in the study done byTalie A, et al.^11^ This was similar to the study done by Malla R, et al. where women admitted with Postpartum Hemorrhage was 18.5%.^7^ Increased morbidity was reported with secondary postpartum haemorrhage in the study done by Hoveyda F, et al. where 63% required surgical evacuation and 17% blood transfusion. Secondary PPH may result in significant morbidity and deserves more attention.^12^

In this study eight patients 7.69% had urinary tract infection, three of them presented with retention of urine. In a study done by Ahnfeldt-Mollerup P, et al. postpartum urinary retention had a reported incidence of 3%.^13^

Postpartum morbidity was higher in caesarean delivery as compared to vaginal delivery in patients delivered in the study hospital. This was similar to the study done by Lydon-Rochelle M, et al. who reported higher readmissions especially due to infectious morbidities.^14^ Twelvepatients, 11.53% were admitted with wound infections in our study. Effective strategies in controlling peripartum infections should be an obstetric priority.

This study was cross sectional study done only in single institute so all the patients with postpartum morbidities might not be included. The result in this study may not show the true picture of the postpartum morbidities in this area.

## CONCLUSIONS

The incidence of postpartum morbidity was found to be 2.96%. Puerperal sepsis, preeclampsia, eclampsia and haemorrhage were the major complications requiring admissions. Ensuring proper postnatal care may contribute to reduction of postpartum maternal complications and help in decline of maternal mortality.

## Conflict of Interest

**None.**
